# CD138 plasma cells may predict brain metastasis recurrence following resection and stereotactic radiosurgery

**DOI:** 10.1038/s41598-019-50298-7

**Published:** 2019-10-07

**Authors:** Michael H. Soike, Jennifer Logue, Shadi Qasem, Ryan T. Hughes, Emory McTyre, Jing Su, Pierre Triozzi, Maurizio Bendandi, Hui-Wen Lo, Tamjeed Ahmed, Stacey S. O’Neill, Waldemar Debinski, Boris Pasche, Kounosuke Watabe, Lance D. Miller, Michael D. Chan, Jimmy Ruiz

**Affiliations:** 1 Hazlerig-Salter Radiation Oncology Center, University of Alabama at Birmingham, Birmingham, AL 35249 USA; 2 University of South Florida Morsani College of Medicine at H. Lee Moffitt Cancer Center, 12902, Tampa, FL 33612 USA; 3 Department of Pathology and Laboratory Medicine, University of Kentucky School of Medicine, Lexington, KY USA; 4 Department of Radiation Oncology, Wake Forest School of Medicine, Winston-Salem, NC 27157 USA; 5 Department of Radiation Oncology, Greenville Health System Cancer Institute, Greenville, SC 29605 USA; 6 Department of Biostatistical Sciences, Wake Forest School of Medicine, Winston-Salem, NC 27157 USA; 7 Department of Medicine (Hematology & Oncology), Wake Forest School of Medicine, Winston-Salem, NC 27157 USA; 8 Department of Cancer Biology, Wake Forest School of Medicine, Winston-Salem, NC 27157 USA; 9 Department of Pathology, Wake Forest School of Medicine, Winston-Salem, NC 27157 USA; 10 W.G. (Bill) Hefner Veteran Administration Medical Center, Cancer Center, Salisbury, NC United States of America

**Keywords:** Cancer, Metastasis

## Abstract

We sought to identify candidate biomarkers for early brain metastasis (BM) recurrence in patients who underwent craniotomy followed by adjuvant stereotactic radiosurgery. RNA sequencing was performed on eight resected brain metastasis tissue samples and revealed B-cell related genes to be highly expressed in patients who did not experience a distant brain failure and had prolonged overall survival. To translate the findings from RNA sequencing data, we performed immunohistochemistry to stain for B and T cell markers from formalin-fixed parffin-embedded tissue blocks on 13 patients. CD138 expressing plasma cells were identified and quantitatively assessed for each tumor sample. Patients’ tumor tissues that expressed high levels of CD138 plasma cells (N = 4) had a statistically significant improvement in OS compared to low levels of CD138 (N = 9) (p = 0.01). Although these findings are preliminary, the significance of CD138 expressing plasma cells within BM specimens should be investigated in a larger cohort. Immunologic markers based on resection cavity analysis could be predictive for determining patient outcomes following cavity-directed SRS.

## Introduction

Approximately 170,000 patients in the United States are diagnosed with brain metastasis (BM) every year^1^. Prognosis can vary considerably depending on factors such as the primary site of disease, histology, burden of systemic disease, and intracranial disease^2–6^. A standard treatment option for limited brain metastases from solid tumor primaries is stereotactic radiosurgery (SRS), which allows for rapid treatment of brain metastases with preservation of cognition, but it is an expensive modality compared to its alternative, whole brain radiation therapy (WBRT) and often requires a specialized center^7–10^.

One limitation of SRS is the potential need and expense of additional treatments for distant brain failure (DBF)^11,12^. Methods of scoring clinical and histopathologic factors have attempted to predict intracranial failure, but these have been met with modest successes, predominantly because of the heterogeneity of the brain metastasis population^13–15^. Developing an accurate biomarker that can help predict for improved DBF or OS in patients with brain metastases treated with SRS would be a valuable tool.

Specific patterns of immune cells within the tumor microenvironment are associated with improved outcome in patients with many types of cancers, regardless of the type of therapy administered^16^.

Tumor infiltrating lymphocytes (TILs) of variable density can be observed in brain metastases and are typically composed of various cell types with a higher fraction of T cells than B cells. The presence of TILs, CD3 and CD8 T cells in specific, has been associated with improved survival as compared to patients with only sparse or scattered TIL infiltration^17^. Furthermore, the Immunoscore, which is based on an automated calculation of the CD3/CD8 ratio among TILs, has been shown to have independent prognostic significance in patients with brain metastases^18^. How TILs influence the response of brain metastasis to SRS is not known.

To identify potential immune biomarkers that could be predictive of outcomes in brain metastasis patients, we performed a retrospective study and tissue analysis of patients who have undergone surgical resection of a brain metastasis followed by SRS. In the initial group of patients, we used microarray to identify immune signatures from resected brain metastasis tissue. We then expanded this microarray profile and sought to characterize immune cells within the brain metastasis tissue with immunohistochemistry associated with favorable outcomes.

## Methods

### Patient selection

This study was approved by the Wake Forest Institutional Review Board IRB00008427. Patients signed informed consent for advanced tissue tumor banking prior to analysis. We selected 8 patients with new brain metastases from various primary tumors who underwent craniotomy and adjuvant SRS. These samples were frozen brain metastasis tissues were collected from patients who consented to the Wake Forest Brain Tumor Center of Excellence Tumor Tissue Bank. These samples were analyzed with RNA sequencing. Six of the original 8 patients had their formalin-fixed paraffin-embedded (FFPE) tissue also evaluated by immunohistochemistry (IHC) and 7 additional patients with available FFPE tissues who had craniotomy and received adjuvant SRS were also included in the study. All methods were performed in accordance with institutional policies, particularly for genomic analysis. A Clinical Laboratory Improvement Amendments certified laboratory was utilized for immunohistochemistry analysis of FFPE tissue with a board certified pathologist (SQ) reviewing the slides.

### Stereotactic radiosurgery

SRS was performed on the Leksell Gamma Knife Model C (Elekta, Stockholm, Sweden) prior to May 2009, and Perfexion after May 2009. Same day headframe fixation was used for immobilization. Patients underwent a contrast-enhanced stereotactic magnetic resonance imaging scan of the brain with headframe in place. The GammaPlan Treatment Planning System (AB Elekta, Stockholm, Sweden) was used to develop the treatment plan. Median marginal dose prescribed was 18–22 Gy and was generally prescribed to the 50% isodose line. The dose selected was based on guidelines previously described by Shaw *et al*.^19^. The targeting of the resection cavity of the metastasis was previously described by Jensen *et al*.^20^.

### Patient follow-up

After adjuvant SRS, patients were followed with repeat MRI approximately 1-2 months later, and then every 3 month basis for 2 years. Distant brain failures (DBF) were determined to be new metastases that developed outside of the prior SRS volume.

### RNA sequencing

**Figure 1:**
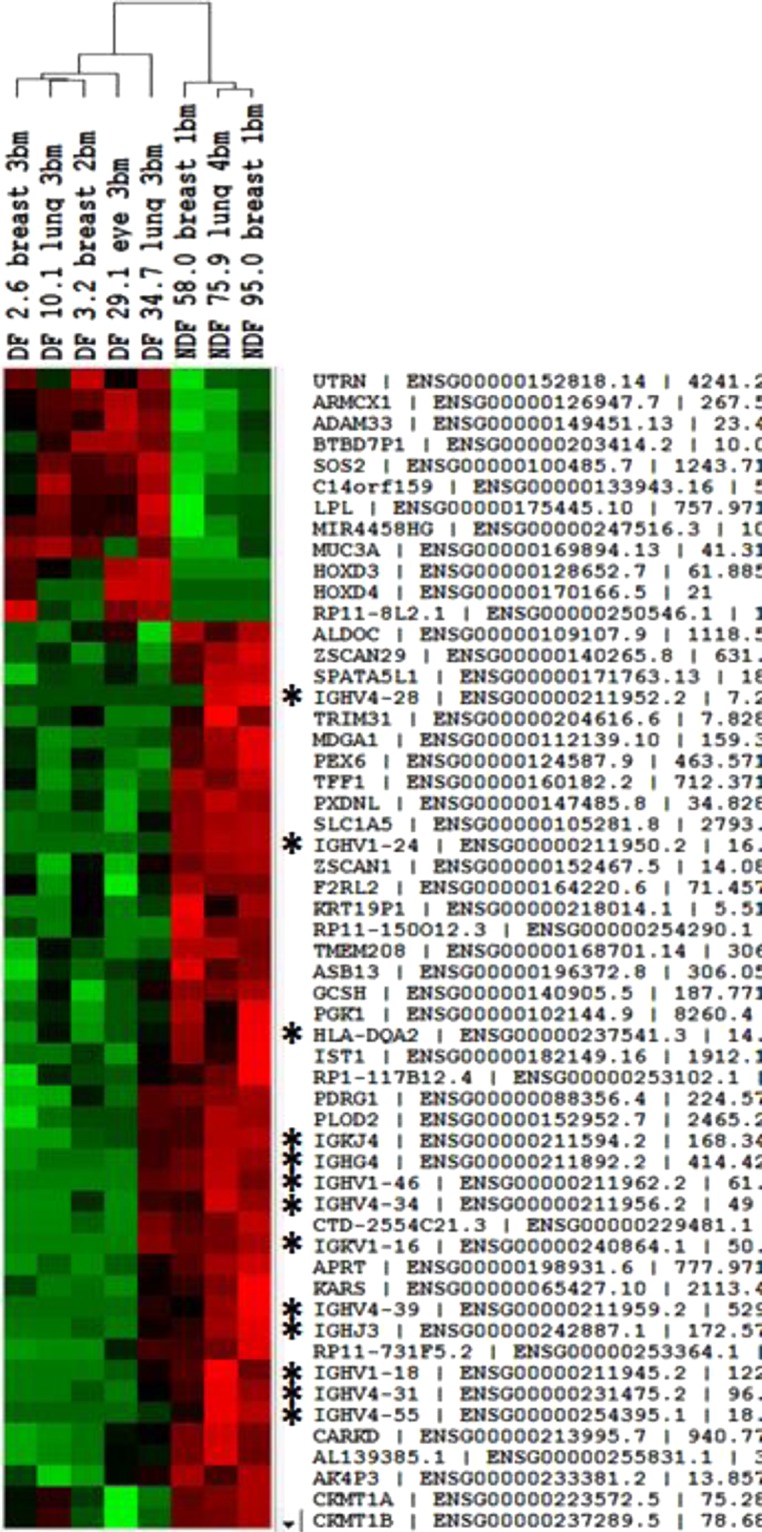
Genomic expression and characterization of eight resected brain metastases. Patients were divided between distant brain failure (DF) and no distant brain failure (NDF) for comparison. Significantly higher gene expression of immune cell populations were identified in the NDF group, as indicated by *.

We identified eight samples from patients who had craniotomy for a new diagnosis of brain metastasis. The frozen brain tumor tissue was assessed using standard sectioning and evaluation for tumor content and viability by a board certified pathologist. Areas with adequate cellularity and viability were selected for testing. Total RNA was purified from the frozen specimens using the RNeasy Plus Micro Kit (Qiagen) with genomic DNA removal. RNA integrity (RIN) was determined by electrophoretic tracing using an Agilent Bioanalyzer. RNAseq libraries were constructed from samples (RIN > 7.0) using the Illumina TruSeq Stranded Total RNA kit with Ribo-Zero rRNA depletion. Indexed libraries were sequenced on an Illumina NextSeq 500 DNA sequencer using 150 × 150-nt paired end reads, generating > 40 million reads per sample (12 samples per flow cell) with > 80% of sequences achieving > Q30 Phred quality scores. Quality of raw sequencing reads were assessed by FASTQC analysis (Babraham Bioinformatics). Sequence reads were aligned using the STAR sequence aligner^21^, and gene counts determined using featureCounts software^22^. Differential gene expression was analyzed using the DESeq2 algorithm^23^. Significant genes were defined as p < 0.05 after adjustment for false discovery (Benjamini-Hochberg). Genes and samples were hierarchically clustered using Pearson correlation as the distance metric and visualized by heatmap analysis (Fig. 1).

### Immunohistochemistry (IHC)

After initial RNA sequencing was performed in a CLIA certified laboratory, IHC was performed on a larger cohort of patients for proteins of interest, 13 in total, including 6 of the previous patients who underwent RNA Seq to correlate the findings from RNA sequencing. Two of the patients in the initial genomic sampling were excluded from IHC analysis because these patients did not meet the criteria of craniotomy followed by adjuvant SRS.

Cases with corresponding formalin-fixed paraffin-embedded tissue were selected for immunohistochemical testing. IHC stains were performed according to validated procedures using DAKO Link 48 autostainer (Agilent technologies, Santa Clara, CA, USA). The tissue was stained for CD20 (DAKO), CD3 (DAKO), CD138-expressing plasma cells (DAKO), Programmed cell death protein-1 (PD1) (Spring Bioscience) and Program death ligand-1 (PDL-1) (Spring Bioscience). Immunohistochemistry stains for T and B cell lymphocyte markers were chosen due to a genomic analysis of a smaller group of heterogeneous patients that revealed high expression of lymphocyte genomic products. The stains were scored on a scale of 0–3 by a board-certified pathologist who was blinded to the immune signature profiles. The number of cells expressing CD138 was quantitatively assessed per 40x high power field. The difference in assessment methods was due to the pattern of distribution of CD138, which had patchy infiltration whereas the other markers showed diffuse infiltration.

### Statistics

Patient outcomes were calculated from time of adjuvant SRS to event using competing risks estimates as previously described by McTyre^24^. Brain metastasis velocity (BMV) was calculated for each patien as described by Farris *et al*.^15^. Kaplan-Meier estimates, and competing risk with subdistribution hazards and figures were calculated using version 3.4.0 software (R Foundation for Statistical Computing, Vienna, Austria).

## Results

### RNA sequencing and IHC

Eight patients with resected brain metastases were found to have several overexpressed B-cell related genes and a lower rate of DBF. Notably, the B-cell genes *HLA-DQA2, IGKJ4, IGKV1-16, IGHG4, IGHJ3, IGHV1-18, IGHV1-24, IGHV1-46, IGHV4-28, IGHV4-31, IGHV4-34, IGHV4-39 and IGHV4-55*, exhibited higher expression compared to patients who experienced a DBF at 58 months post-treatment (Fig. 1).

Thirteen patients were analyzed with IHC. This group of 13 patients had a median follow up of 29.5 months [Interquartile range (IQR) 11.1–37.5]. Histologies represented were melanoma (N = 2), Breast (N = 4), non-small cell lung cancer (N = 6), and small cell lung cancer (N = 1) (Table 1). The patient with small cell lung cancer had received prophylactic cranial irradiation 2 years prior to craniotomy, no other patients received whole brain radiation prior to craniotomy. Twelve of the 13 patients received steroids prior to craniotomy for brain metastasis; it remained unknown if the remaining patient received steroids. The median KPS was 90 [IQR 70–90]. At time of analysis, 2 local failures had occurred, 8 distant brain failures were observed, 4 patients experienced leptomeningeal failure, 4 patients received salvage WBRT, and 9 patients had died.

**Table 1:** Patient characteristics and CD138 expression. Brain Metastases (BM), Intracranial (IC), Brain metastasis velocity (BMV), Small cell lung cancer (SCLC), Non-small cell lung cancer (NSCLC), Adenocarcinoma (adeno), Not otherwise specified (NOS), Distant Brain Failure (DBF), Local Failure (LF), No event (NE).

Histology	Number of BM treated at adjuvant SRS	CD138	First IC Event	Months to 1st IC event	BMV	Overall survival (months)
Melanoma	3	5	DBF	29	1.6	42
Melanoma	2	7	DBF	8	26	15
SCLC	1	22	DBF	2.5	26	12
Breast, Her2+	3	0	DBF	2	3.9	25
Breast, Her2−	1	10	NE		0	77
Breast, Her2−	1	1	LF + DBF	3.2	0	22
NSCLC, adeno	3	36	LF + DBF	7.3	3.5	11
NSCLC, adeno	4	60	DBF	6.5	5.6	9
NSCLC, NOS	5	1	NE		0	4
Breast, Her2+	1	191	NE		0	125, alive
NSCLC, adeno	1	247	NE		0	29, alive
NSCLC, NOS	3	143	NE		0	25, alive
NSCLC, NOS	3	336	DBF	26	0.3	105, alive

**Figure 2:**
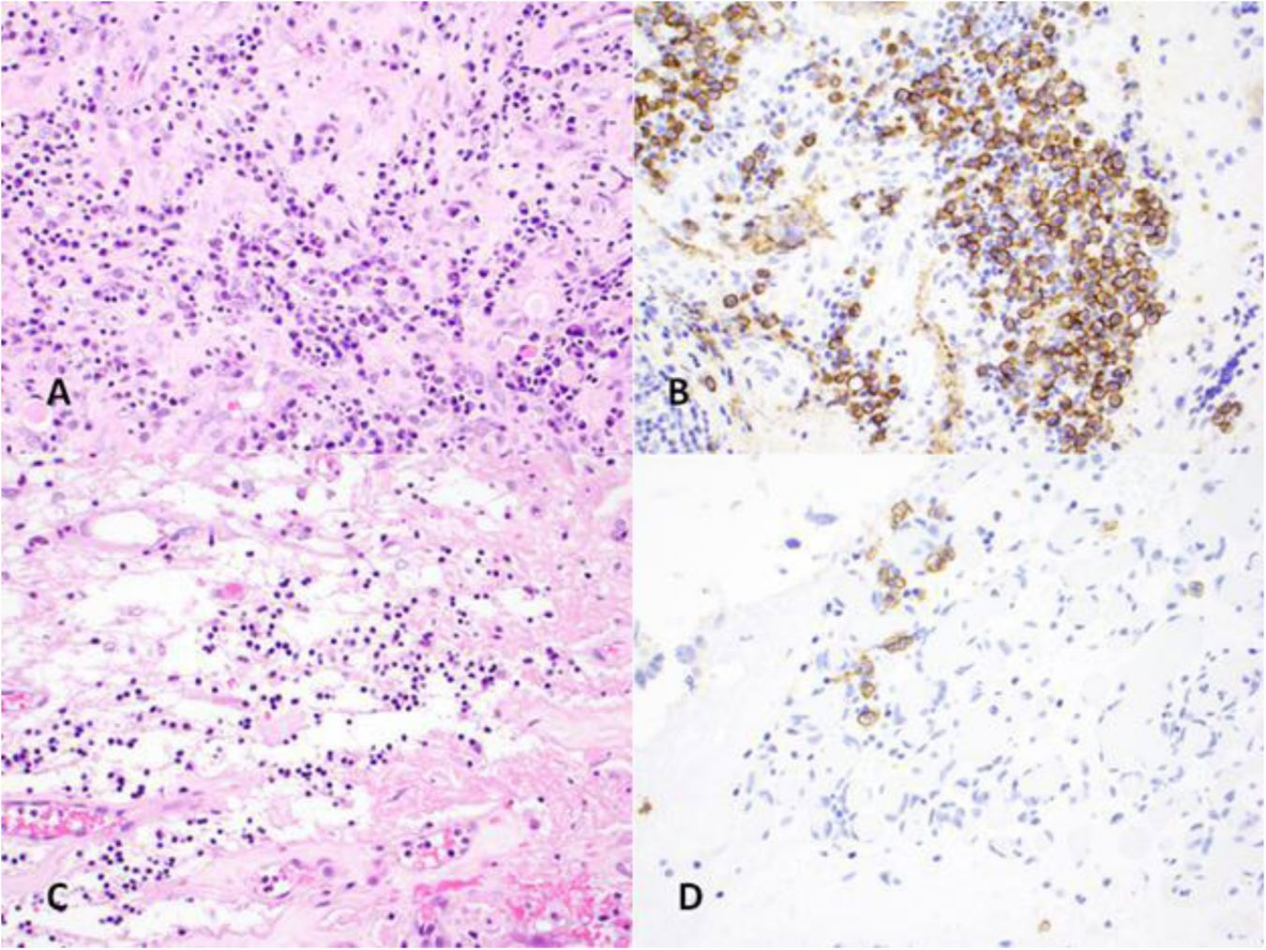
(**A**) and (**B**), a case with high plasma cells. (**C**,**D**) A case with low plasma cells. [(**A**) and (**C**), hematoxylin and eosin. (**B**) and (**D**), CD138 immunohistochemical stain. All images taken at 40X magnification].

PD-L1 was highly expressed in three patients and PD1 overexpressed in 1 patient. High expression of CD3 was observed in 9 patients and high expression of CD20 was observed in 4 patients. CD138 was quantitatively assessed and demonstrated bimodal distribution pattern. Nine patients had CD138 cell counts of 60 or less (range 0–60) and 4 patients had CD138 cell counts of greater than 140 (range 143–247). Figure 2 is a representative picture of H&E stains.

**Figure 3:**
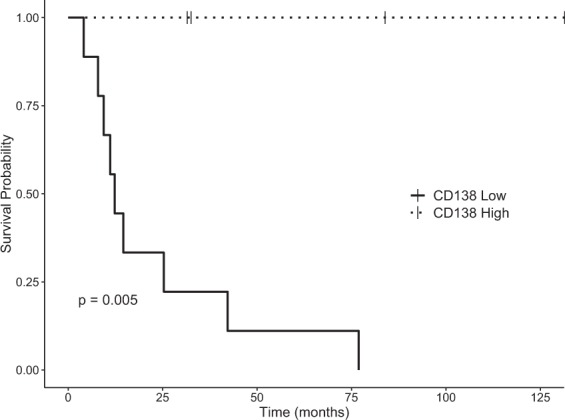
Kaplan Meier estimates of overall survival for patients with stratified by high or low CD138 positivity.

Of the patients with high expression of CD138, no deaths have been observed, no local failures, and one DBF has been recorded. All patients with high expression of CD138 were still alive. Of these patients, 3 had NSCLC and 1 had breast cancer. The median OS in patients with low expression of CD138 was 14.7 months vs not reached for high expression of CD138 (Log Rank p = 0.01) (Fig. 3). Competing risk for time to first intracranial event between CD138 high expression vs low expression did not reach statistical significance (P = 0.11). Univariate analysis of other IHC stains (CD3, CD20, PD-1, PDL1) did not reveal a statistically significant association between cell marker expression and OS or intracranial failure.

## Discussion

For patients who underwent craniotomy followed by adjuvant SRS for brain metastases, high levels of CD138 lymphocytes within craniotomy samples were associated with improved survival compared to patients with low levels of CD138 lymphocytes. CD138 is a plasma cell marker and a proteoglycan, syndecan 1, which is expressed by solid tumor cells and plasma cells. When expressed by tumor cells, CD138 has been associated with metastasis and poor survival^25,26^. However, the infiltration of CD138 plasma cells in solid tumors has been associated with longer survival in patients with ovarian, gastroesophageal, NSCLC, and colon cancers^27^. These effects might be augmented by the localization within tumor, which would enable high concentrations of antibody to accumulate locally. Antibodies could opsonize tumor antigens, thereby facilitating antigen presentation and broadening of T-cell responses^28–30^. Moreover, antibodies could mediate direct antitumor effects by binding to and disrupting the function of their cognate antigens, activating the complement pathway, and/or triggering antibody-dependent cellular cytotoxicity^31^.

Unlike biomarkers that predict for brain metastasis-specific response to a systemic agent, a biomarker with the capability of predicting SRS outcomes in patients with brain metastases from different primary tumors would have significant clinical potential given the current dilemma by which patients are selected for upfront SRS *versus* upfront WBRT. Several statistical models have been developed in order to help triage patients, but the ability of validate them has been somewhat questionable^14,32^. A major issue with validation of predictive models for brain metastases has been the biological heterogeneity of brain metastases and the fact that brain metastases of different primary tumors have distinct natural histories due to variations in systemic disease burden and control^5,33^. The discovery of the immunotype changes that drive these biological differences will hopefully help to improve the predictability of brain metastasis outcomes moving forward.

This study is limited by a small sample size, retrospective nature, and requires further validation in a large cohort of patients prior to utilizing CD138 as a biomarker for clinical practice. However, the identification of an immune marker within resected brain metastasis tissue that translates into improved survival outcomes is an exciting finding and warrants further investigation.

## Conclusion

Patients with high levels of CD138 expressing plasma cells may have improved OS compared to patients with low levels of CD138 with a trend towards fewer intracranial failures. The results are hypothesis generating and CD138 expression should be investigated in a larger cohort of patients with resected brain metastasis tissue.
